# A surprising diagnosis of paracoccidioidomycosis from routine bacterial culture of chronic finger lesions

**DOI:** 10.1128/asmcr.00133-25

**Published:** 2025-11-05

**Authors:** Nikita Jaggernauth, Juan Pineda-Reyes, Claire Zurlo, Devin M. Weber, Matthew A. Pettengill, Nathan P. Wiederhold, Connie Cañete-Gibas, Courtney E. Comar

**Affiliations:** 1 Division of Infectious Diseases and Environmental Medicine, Department of Internal Medicine, Thomas Jefferson University 6559, Philadelphia, Pennsylvania, USA; 2 Department of Pathology and Genomic Medicine, Thomas Jefferson University 6559, Philadelphia, Pennsylvania, USA; 3 Department of Internal Medicine, Thomas Jefferson University Hospital 23217, Philadelphia, Pennsylvania, USA; 4 Department of Pathology and Laboratory Medicine, University of Texas Health Science Center at San Antonio 14742, San Antonio, Texas, USA; Rush University Medical Center, Chicago, Illinois, USA

**Keywords:** fungal infections, endemic mycoses

## Abstract

**Background:**

Paracoccidioidomycosis is a systemic mycosis endemic to Central and South America. Here, we describe an unusual case of an elderly female with cutaneous paracoccidioidomycosis and comorbidities limiting the use of first-line therapeutic agents.

**Case Summary:**

An 89-year-old female with a past medical history of advanced systolic heart failure presented with decompensated heart failure along with chronic, progressive ulcerating lesions involving multiple fingers, unresponsive to antibiotic therapy. A biopsy showed evidence of yeast on pathology and samples grew *Paracoccidioides brasiliensis* within 5 days on routine bacterial culture. Further history revealed that she lived in Trinidad and Tobago for periods during her childhood and had visited Ecuador for 2 weeks decades prior. Due to her history of advanced congestive heart failure on dobutamine and ventricular arrhythmias, first-line therapy with itraconazole was contraindicated. Sulfamethoxazole-trimethoprim was initiated; however, she developed progressive renal dysfunction and hyperkalemia attributed to sulfamethoxazole-trimethoprim, necessitating a change in therapy. The patient was ultimately started on isavuconazole, which has been shown to be effective in the treatment of *Paracoccidioides* in a small phase 3 trial. Unfortunately, we were unable to observe clinical response to isavuconazole in our patient due to her demise in the setting of advanced heart disease.

**Conclusion:**

This case highlights the variable nature of paracoccidioidomycosis, with our patient having a prolonged latency prior to development of clinical signs and symptoms, an unusual diagnosis by growth in routine bacterial culture, and the need for further research in alternative therapeutic options.

## INTRODUCTION

Paracoccidioidomycosis is a mycosis caused by the thermally dimorphic fungi, *Paracoccidioides* species, endemic to Central and South America with rare cases reported in the United States (US). Diagnostic options for *Paracoccidioides* infections are limited in the US, primarily relying on culture or histopathologic findings. Here, we present a case of paracoccidioidomycosis diagnosed by growth of the yeast form in bacterial culture in an elderly patient with chronic, cutaneous lesions who had a remote travel history with no sustained endemic exposure.

## CASE PRESENTATION

An 89-year-old female with a history of cardiac amyloidosis complicated by advanced systolic heart failure, on prolonged intravenous inotropes, secondary to cardiac amyloidosis, presented with an 8-week history of painful ulcerative lesions on multiple fingers.

The patient noted a 1-year history of intermittent ulcers involving multiple fingers with spontaneous resolution. Eight weeks prior to admission, she noted the onset of painful nodules involving multiple fingers, which ulcerated over time. She denied preceding trauma, erythema, purulence, fever, or chills. At her long-term care facility, she received daily wound care and a 2-week course of empiric vancomycin and cefepime with some transient improvement.

Of note, the patient was born in New York City, grew up in Trinidad and Tobago, and over her lifetime, spent time between the United States, Trinidad and Tobago, lived in China for several months, and had a distant history (unknown dates but decades prior as a young adult) of travel to Ecuador for 2 weeks. She had been living permanently in the US in a skilled nursing facility for 18 months before her presentation.

**Fig 1:**
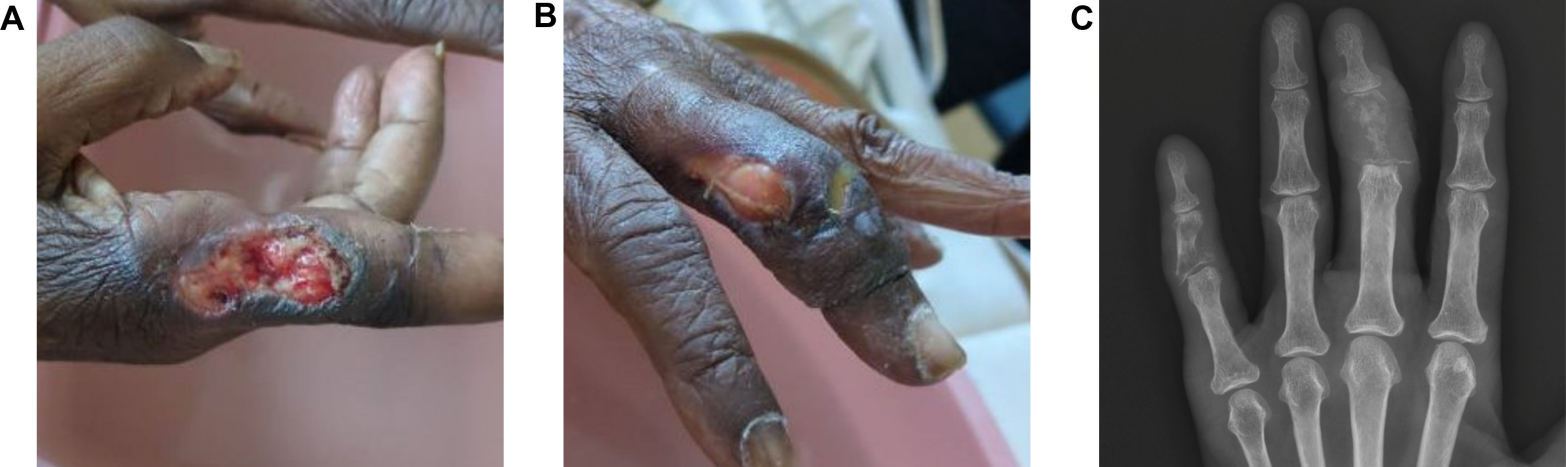
Clinical images. (**A**) Right hand ulcerative lesion on hospital admission. (**B**) Left hand ulcerative lesion on hospital admission. (**C**) Initial radiograph of the left hand showing osteomyelitis of the middle phalanx.

On physical examination, there were four distinct ulcerative lesions on her left fifth and third digits and her right fifth and second digits (Fig. 1A and B). The ulcers were clean based with hyperpigmented borders. An X-ray of the left hand showed erosions in the middle phalanx of the third finger with associated soft tissue swelling and areas of demineralization in the middle phalanx of the fifth finger (Fig. 1C). An X-ray of the right hand revealed demineralization and erosions in the proximal phalanx of the second digit, with surrounding soft tissue swelling and a small radiolucent area in the proximal phalanx of the third digit. A punch biopsy of the right fifth digit was performed by a dermatologist. She was also noted to have hyperpigmented lesions on her nose, and a shave biopsy was performed.

Initial laboratory studies on presentation included a normal white blood cell count of 4.3 B/L (reference range 3.7–10.5 B/L), an elevated C-reactive protein of 2.6 mg/dL (reference range ≤ 0.5 mg/dL), a negative HIV Ab/Ag test, and an erythrocyte sedimentation rate of 31 mm/h (reference range 0–30 mm/h). Routine blood cultures (aerobic and anaerobic) collected on admission were negative.

**Fig 2:**
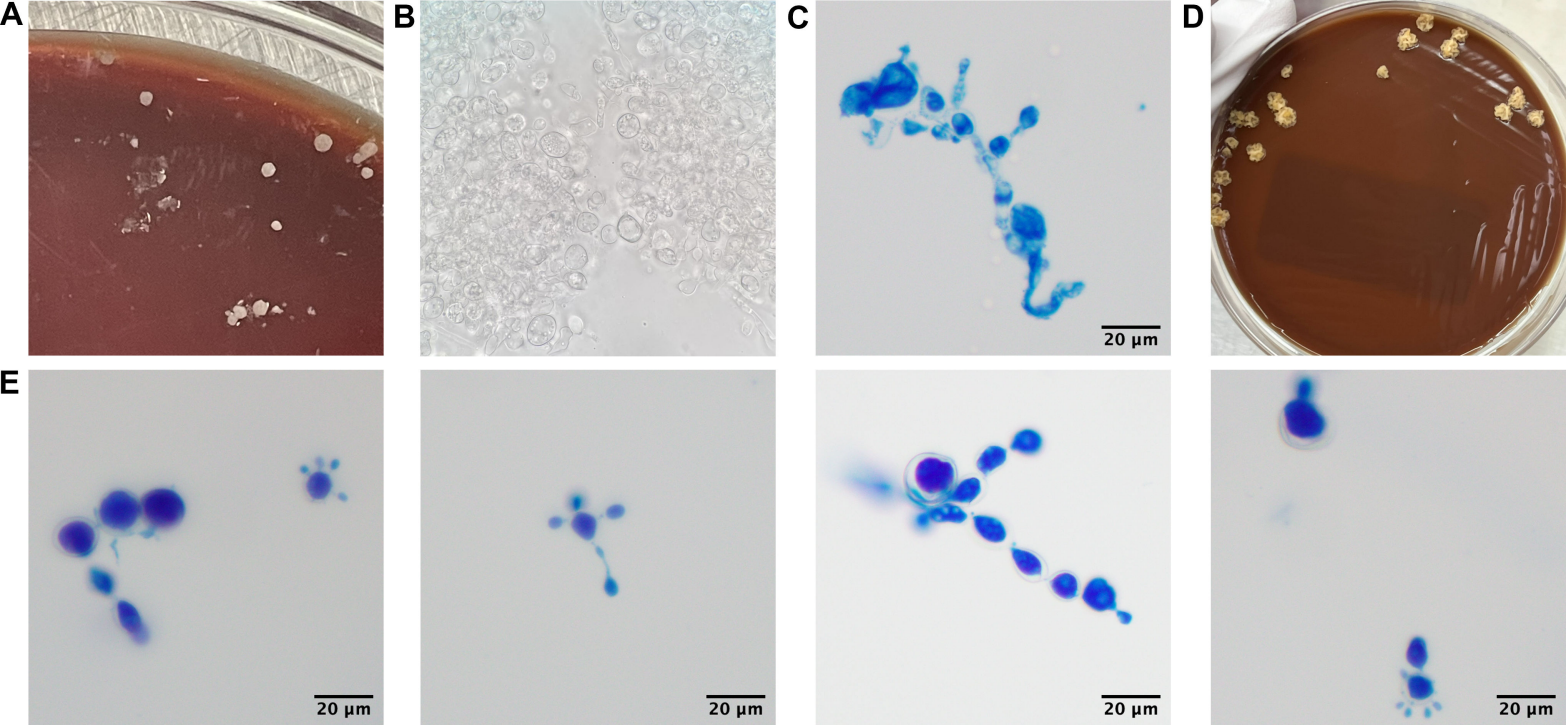
Macroscopic and microscopic morphologies of *Paracoccidioides brasiliensis* isolate. (**A**) Colony morphology of isolate on blood agar plate on day 10 of aerobic bacterial culture (35°C). (**B**) Wet mount (1,000×) from isolate on day 10 of aerobic bacterial culture. (**C**) Lactophenol cotton blue print (400×) of isolate subculture from BHI after 10 days of incubation at room temperature. (**D**) Colony morphology of isolate on chocolate agar after 5 weeks. (**E**) Lactophenol cotton blue prints (400×) of isolate after 5 weeks, demonstrating yeast forms.

A biopsy specimen from the right fifth digit was submitted for pathology along with routine bacterial and mycobacterial cultures. The gross description was reported as an ulcer. No organisms were noted initially on stains performed on pathology specimens (Gram, Periodic acid–Schiff-diastase [PAS-D], Kinyoun Acid Fast). On day 5 of the bacterial culture, a small, dry, white colony was observed on the 5% sheep blood agar aerobic plate (35°C). The initial Gram stain was reported as a Gram-positive rod. Identification was attempted by MALDI-TOF mass spectrometry (Bruker MS), which was unrevealing (no peaks, MBT-CA Reference Library). Ongoing growth of multiple, dry, crumbly, white colonies was seen on both blood and chocolate agar at 35°C (Fig. 2A). Attempted identification by MALDI-TOF MS failed again on day 7. A repeat Gram stain of the isolate on day 8 demonstrated yeast-like structures. A wet mount (Fig. 2B) and calcofluor white stain were performed from the isolate on day 9, which revealed large (>15 μm), irregular yeast cells, with some cells demonstrating multiple buds and hyphae concerning for possible *Paracoccidioides*, although the time of growth in culture seemed too rapid for what would typically be expected for *Paracoccidioides*.

**Fig 3:**
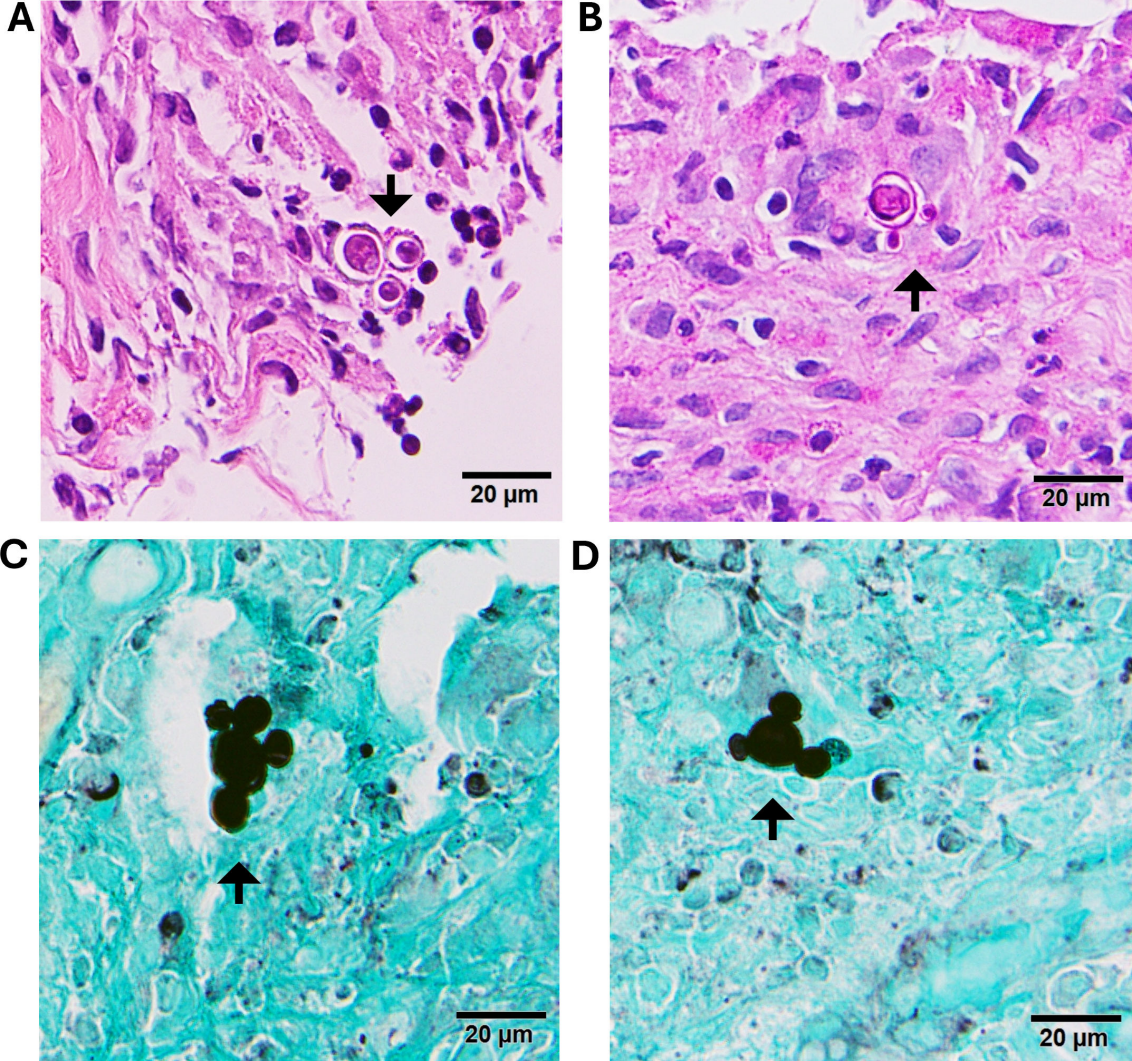
Histopathology of right digit biopsy demonstrating yeast with multiple buds at 400× seen on H&E (**A**), PAS-D (**B**), and GMS (**C,D**).

Growth of a fungal organism prompted a review of the biopsy specimen. Round, budding yeast were observed on the pathology stains: H&E, PAS-D, and Grocott–Gömöri’s methenamine silver (GMS) (Fig. 3). Some yeast forms demonstrated multiple buds. The histopathology of the lesion on the left nasal ala showed granulomatous dermatitis with pseudoepitheliomatous hyperplasia. Subsequent review of the GMS stain demonstrated rare yeast forms.

Further testing included negative serum *Blastomyces, Histoplasma,* and *Coccidioides* antibody immunodiffusion testing and (1→3)-β-D-Glucan. Quantitative *Histoplasma* antigen from the urine was positive at 0.33 ng/mL (reference range >0.20 ng/mL positive). The result report noted cross-reactions occur with *Blastomyces* spp., *Coccidioides* spp., and *Paracoccidioides brasiliensis*.

The isolate was submitted to a reference laboratory (Fungus Testing Laboratory at the University of Texas Health Science Center at San Antonio) for identification. ITS and D1/D2 of the nuclear ribosomal DNA were sequenced (GenBank Accession numbers: ITS—PX353476, D1/D2—PX353475) to confirm the identity of the isolate as *Paracoccidioides* species. BLASTn searches (accessed on 25 March 2024, https://blast.ncbi.nlm.nih.gov/Blast.cgi) confirmed that the isolate was *Paracoccidioides* species in the *P. brasiliensis* species complex. As shown by the BLASTn results (Table 1), it was not possible to distinguish *P. brasiliensis* from *P. venezuelensis* and *P. restrepoana* using ITS and D1/D2. Sequencing another gene locus (the ADP-ribosylation factor) was attempted, but sequencing failed. At the time of the case, the isolate was therefore identified as *P. brasiliensis* complex. There have been taxonomic updates since the conclusion of the case that are further addressed in the discussion, which would update the identification to *P. brasiliensis*. Serology by immunodiffusion for *Paracoccidioides* (performed at the Centers for Disease Control) was positive with a band of identity for *Paracoccidioides* antigen present.

**TABLE 1:** Results from sequencing identification of isolate

Reference strains (1–3)	% Identity (query coverage; GenBank accession)	
	ITS (PX353476)	D1/D2 (PX353475)
*Paracoccidioides brasiliensis* PB18**^T^**	99.80% (88%; KT155977)	100% (100%; XR_007137646)
*Paracoccidioides brasiliensis* CBS 372.73	99.84% (98%; MH860706)	99.83% (94%; MH872413)
*Paracoccidioides restrepoana* EPM83 PS3	99.61% (78%; KJ540978)	NA^*a*^
*Paracoccidioides restrepoana* Pb60855	99.45% (84%; KJ540978)	NA
*Paracoccidioides venezuelensis*	NA	NA
*Paracoccidioides americana* EPM 194 PS2	99.14% (92%; KT155971)	100% (92%; KT155326)
*Paracoccidioides americana B23 = Pb4*	98.94% (86%; AY374336)	NA
*Paracoccidioides ceti* MSC B92-932**^T^**	98.10% (95%; NR_177553)	NA
*Paracoccidioides lutzii* ATCC MYA 826**^T^**	97.76% (75%; NR_155022)	99.35% (100%; XR_001551861)

^*a*^ NA, sequence not available for this strain.

The patient received empiric intravenous vancomycin (1 g Q24 dosed by level) and cefepime (1 g Q24 renally dosed) on admission, which was discontinued after evaluation by the infectious disease team. Due to her advanced heart failure on dobutamine, prolonged QTc interval, and risk of arrhythmias, she was not a candidate for first-line therapy with itraconazole (or other azoles except isavuconazole) and was ultimately started on sulfamethoxazole-trimethoprim 400–80 mg (SMX-TMP) twice daily by mouth. However, the patient developed renal injury and hyperkalemia within 14 days, necessitating a change to isavuconazonium sulfate (isavuconazole prodrug) 372 mg by mouth daily. Unfortunately, she passed away after over 2 months of hospitalization due to progressive heart failure.

## DISCUSSION

*Paracoccidioides* species are thermally dimorphic fungi and the causative agents of paracoccidioidomycosis, a fungal disease endemic to humid regions surrounding forests, rivers, and agricultural fields in South and Central America, with most cases and literature originating from Brazil (4–6). Notably, paracoccidioidomycosis is not endemic in Trinidad and Tobago, where this patient lived for several years. Infection occurs primarily through inhalation of environmental conidia. The role of cutaneous inoculation as a mechanism of infection is still a matter of debate. Risk factors include exposure to soil and dust related to outdoor occupations, such as coffee and tobacco cultivation (4, 6). Global warming, changing agricultural practices, inhabitation of unexplored territories, and deforestation contribute to the increasing incidence of paracoccidioidomycosis (4, 6).

*Paracoccidioides* species are thermally dimorphic fungi. Growth of mycelial forms is slow, requiring 2 to 6 weeks of incubation at 25°C, and colonies have a whitish, cottony, irregular appearance with short aerial mycelia (7, 8). The yeast form is observed in infected host tissue and can be recovered in culture at 35°C–37°C. Microscopically, the yeast phase of *Paracoccidioides* has multiple narrow-based budding daughter cells, classically resembling the spokes of a mariner’s wheel. However, they can also present as single cells with one bud or forming short chains (7, 8). Yeast grow as tan to cream-colored wrinkled colonies and grow more rapidly than the mycelial form within a week (7, 9). *Paracoccidioides* grow on enriched media, including brain heart infusion, Pine’s, or Kelly’s agar (10, 11). *Paracoccidioides* species can be separated into cultivatable (*P. americana*, *P. brasiliensis*, and *P. lutzii*) and uncultivatable (*P. ceti* and *P. lobogeorgii*) species (12). *P. brasiliensis* is often cited in literature as a complex, including cryptic species *P. brasiliensis, P. restrepoana,* and *P. venezuelensis*. However, the current taxonomic status does not recognize *P. restrespoana and P. venezuelensis* as valid species (12).

Diagnostics for *Paracoccidioides* in the United States (US are limited. Diagnosis is primarily made by culture and/or direct observation by microscopy of the characteristic yeast form in specimens. Identification is typically performed by demonstrating thermal dimorphism. Identification could also be performed by sequencing identification. Literature on the use of MALDI-TOF for the identification of *Paracoccidioides* is limited, and it is not found in FDA-cleared databases for MALDI-TOF in the US. Additional diagnostics include laboratory-developed tests for serology for *Paracoccidioides*, although in the US this testing is only offered at select reference laboratories. There is no specific antigen testing available; however, cross-reactivity may occur with other fungal antigen tests, such as *Histoplasma*, which we believe was the reason for the elevated *Histoplasma* urine antigen in our patient. Data on the use of other fungal antigen tests (1-3-β-D-glucan or galactomannan) are limited, although a recently published study demonstrated patients with paracoccidioidomycosis had positive 1-3- β-D-glucan serum testing (13).

Following exposure, over 95% of people remain asymptomatic and will have a positive paracoccidioidin skin test; however, unlike histoplasmosis, there is typically no evidence of pulmonary calcifications or nodules on imaging. In symptomatic cases, two clinical forms have been described, the acute or subacute form (juvenile) and the chronic (adult) form, as proposed at the Encuentro Internacional de la paracoccidioidomycosis, Medellín, 1986 (14). The juvenile form typically affects patients under 30 years old, represents approximately 10% of cases, typically involves the monocyte-macrophage system (lymph nodes, liver, spleen, and bone marrow) and tends to be a more severe and progressive form of disease. Symptom onset usually occurs within 45 days following exposure (15). Presentation can be variable, including fever, weight loss, malaise, skin lesions, lymphadenopathy, draining fistulae, and hepatosplenomegaly.

The adult or chronic form is seen in 80% to 90% of cases, is more frequent in adult men, typically involves the lungs, and represents reactivation of disease from prior exposure. Symptoms are non-specific and include fever, malaise, cough, and dyspnea. One-third of patients present with chronic pulmonary sequelae, such as pulmonary fibrosis, bullae, and pulmonary hypertension. In over 50% of cases, there is hematogenous spread with mucosal involvement, including the GU system, larynx, and oropharynx, leading to characteristic oral mulberry-like erosions (Aguiar-Pupo stomatitis). Cutaneous lesions are seen in 25% of cases, with a variable presentation, including ulcers, crusted papules, nodules, and plaques (16).

*Paracoccidioides* species are sensitive to most antifungal agents, including amphotericin B, the azoles, terbinafine, and even sulfonamides, such as SMX-TMP (17). Of these agents, itraconazole has been studied the most extensively and is typically the first-line agent for mild to moderate disease with response rates of 85%–90% (18). Alternative azoles that have been studied include voriconazole, which showed similar efficacy compared with itraconazole, though data are limited (18). Isavuconazole was evaluated in a small study in which 10 patients received primary treatment with isavuconazole for an average of 180 days (range 27 to 182) with clinical response observed in eight patients (one complete, seven partial), while the other two patients died (19). SMX-TMP (800–160 mg bid or tid) has been widely used as an alternative to itraconazole (20); however, there are no randomized trials evaluating its efficacy vs itraconazole. Several retrospective studies have found similar outcomes when compared to itraconazole, although a longer treatment period, more frequent side effects, and early discontinuation were observed with SMX-TMP (21–23). In severe paracoccidioidomycosis (hypotension, respiratory failure, severe malnutrition, central nervous system involvement), a 2- to 4-week course of amphotericin B is recommended initially (21).

The duration of treatment is ill-defined, as no randomized trials have compared different treatment durations and is guided by clinical and radiologic resolution along with reduction in antibody titers (24). Azoles are typically continued for 6 to 12 months, while SMX-TMP requires a longer period of treatment to prevent relapse, usually ≥2 years. Patients with severe disease often require longer courses of therapy (>2 years), even when itraconazole is used. Patients with central nervous system (CNS) involvement and immunocompromised hosts may also need longer therapy. Serial serologic testing by quantitative immunodiffusion should be performed prior to the initiation of therapy, after 3 months of therapy, and every 6 months thereafter until the completion of therapy (24). Clinical improvement with resolution of signs and symptoms typically occurs within 1–8 weeks after initiating treatment, while radiographic improvement should occur after 3–12 months.

Unique features of this case included the patient’s brief, remote (several decades prior) travel to an endemic country and the isolation of the yeast form of *Paracoccidioides* within 5 days on routine bacterial culture. At the time of the case, the pathogen was identified by sequencing as *P. brasiliensis* complex (unable to distinguish between *P. brasiliensis, P. venezuelensis,* and *P. restrepoana*); however, since conclusion of the case, the isolate would be identified as *P. brasiliensis* due to changes in taxonomic validity and exclusion of *P. venezuelensis* and *P. restrepoana* from the *P. brasiliensis* complex (12). Another unique feature of the case included clinical management. The patient was unable to be treated with first-line therapy; therefore, SMX-TMP was initiated. Due to additional complications, she was subsequently started on isavuconazole. Unfortunately, due to her passing shortly after initiating isavuconazole, we were unable to observe her clinical response to contribute to the literature supporting its use.
